# SND1 promotes Th1/17 immunity against chlamydial lung infection through enhancing dendritic cell function

**DOI:** 10.1371/journal.ppat.1009295

**Published:** 2021-02-26

**Authors:** Xinting Wang, Chunyan Zhang, Shuhe Wang, Rasheduzzaman Rashu, Rony Thomas, Jie Yang, Xi Yang

**Affiliations:** 1 Department of Immunology, University of Manitoba, Winnipeg, Canada; 2 Key Laboratory of Immune Microenvironment and Disease, Ministry of Education, Key Laboratory of Cellular and Molecular Immunology in Tianjin, Department of Biochemistry and Molecular Biology, Excellent Talent Project, Department of Immunology, School of Basic Medical Sciences, Tianjin Medical University, Tianjin, China; University of California, Davis, UNITED STATES

## Abstract

To date, no reports have linked the multifunctional protein, staphylococcal nuclease domain-containing protein 1 (SND1), to host defense against intracellular infections. In this study, we investigated the role and mechanisms of SND1, by using SND1 knockout (SND1^-/-^) mice, in host defense against the lung infection of *Chlamydia muridarum*, an obligate intracellular bacterium. Our data showed that SND1^-/-^ mice exhibited significantly greater body weight loss, higher organism growth, and more severe pathological changes compared with wild-type mice following the infection. Further analysis showed significantly reduced *Chlamydia*-specific Th1/17 immune responses in SND1^-/-^ mice after infection. Interestingly, the dendritic cells (DCs) isolated from SND1^-/-^ mice showed lower costimulatory molecules expression and IL-12 production, but higher IL-10 production compared with those from wild-type control mice. In the DC-T cell co-culture system, DCs isolated from SND1^-/-^ infected mice showed significantly reduced ability to promote *Chlamydia*-specific IFN-γ producing Th1 cells but enhanced capacity to induce CD4^+^T cells into Foxp3^+^ Treg cells. Adoptive transfer of DCs isolated from SND1^-/-^ mice, unlike those from wild-type control mice, failed to protect the recipients against challenge infection. These findings provide *in vivo* evidence that SND1 plays an important role in host defense against intracellular bacterial infection, and suggest that SND1 can promote Th1/17 immunity and inhibit the expansion of Treg cells through modulation of the function of DCs.

## Author summary

Since the initial study from our group reported that SND1 is able to hijack nascent MHC-I heavy chain in tumor cells, thereby impairing the proper assembling of MHC-I and sensitizing tumor cells to a diminished immune surveillance with abolished antigen presentation to cytotoxic CD8+ T cells, large attention has been drawn to its importance in host immunity. To date, no reports have been linked SND1 to immune response against bacterial infection. In this study, we investigated the role and mechanisms of SND1 in chlamydial lung infection by using SND1 knockout mice. We found that SND1 knockout mice showed significantly delayed clearance of bacteria and more severe disease. More importantly, we found that the SND1 deficiency led to alteration of phenotype and function of dendritic cells, which is associated with a failure in the development of protective Th1/17 immunity. These data indicate that SND1 protein plays an important role in host defense against chlamydial infection, at least partially through modulating dendritic cell function.

## Introduction

*Chlamydia trachomatis*, an obligate intracellular bacterium, afflicts people with a broad range of diseases, including sexually transmitted diseases and pneumonia[1]. *Chlamydia muridarum* (Cm), a natural chlamydial strain of murine, has been commonly used in murine models of respiratory and genital tract infections[2]. Recent studies have shown the implications of Th1 immunity particularly IFN-γ production for host defense against chlamydia[3–5]. More recently, it was found that IL-17/Th17 responses are also significant for protection against chlamydial lung infection[6,7]. Accumulating effort has been made to elucidate the mechanisms underlying varioius patterns of cytokine responses in chlamydial infection and to enhance the type 1 and/or Th17 immune responses against protection.

DCs are the primary antigen-presentation cells (APCs) of the immune system and have a key role in both sensing pathogens and tuning the immune responses[8,9]. They consist of various subtypes and are classified on the basis of their phenotype, location, and function[10,11]. Conventional DCs (cDCs) mainly reside in the lymphoid tissues such as thymus, spleen, and secondary lymph nodes (LNs). These conventional DCs exhibit higher levels of MHC-II and CD11c and can be subsequently divided into CD8*α*^+^ and CD8*α*^−^ DCs in mice. When compared with CD8*α*^+^ DCs which more commonly induce Th0 cells to elicit Th1 response, CD8*α*^−^ DCs more likely induce Th2 responses[12–14]. In addition, cDCs in the nonlymphoid tissues including the intestine and the lung are composed of two major subsets: CD103^+^ and CD11b^hi^ DCs. Interestingly, CD103^+^ DC in the nonlymphoid organs such as gut, lung, and skin form a combined subset, which is developmentally related to the CD8^+^ cDC in lymphoid organs[13,15,16].

SND1 (staphylococcal nuclease domain containing 1), also known as Tudor staphylococcal nuclease (Tudor-SN), is a highly conserved and ubiquitously expressed multifunctional protein. It comprises a tandem repeat of four staphylococcal nucleases (SN)-like domains (referred to as SN domains) at the N terminus and a fusion of a Tudor domain with a partial SN domain at the C terminus (referred to as TSN domain). SND1 regularly localized in the cytoplasm unless under certain circumstances such as stress or signaling induction in which it may shuttle between nucleus and cytoplasm[17]. SND1 plays important roles in the early stage of DDR (DNA damage response) in a poly-ADP-ribosylation-dependent manner via interaction with PARP-1 by SN domain[18]. SND1, being indispensable for normal development, promotes cell cycle progression by facilitating E2F-1-mediated gene transcription[18,19]. Global transgenic SND1 in mice is prevented from obesity-induced insulin resistance whereas its increased expression is closely associated with various cancers[20–22]. Our recent study has revealed that SND1, as an oncogene, is able to hijack nascent MHC-I heavy chain in tumor cells, thereby impairing the proper assembling of HC and sensitizing tumor cells to a diminished immune surveillance with abolished antigen presentation to cytotoxic CD8+ T cells[23], which is the initial report that links SND1 to immune system.

To date, no reports have been linked SND1 to immune response against bacterial infection. In this study, we investigated the role and mechanisms of SND1 in chlamydial lung infection by using SND1 knockout mice. We found that SND1 knockout mice showed significantly delayed clearance of bacteria and more severe disease. More importantly, we found that the SND1 deficiency led to alteration of phenotype and function of DC, which is associated with a failure in the development of protective Th1/17 immunity. These data indicate that SND1 protein plays an important role in host defense against chlamydial infection, at least partially through modulating DC function.

## Results

### SND1 contributes to protection against chlamydial lung infection

**Fig 1:**
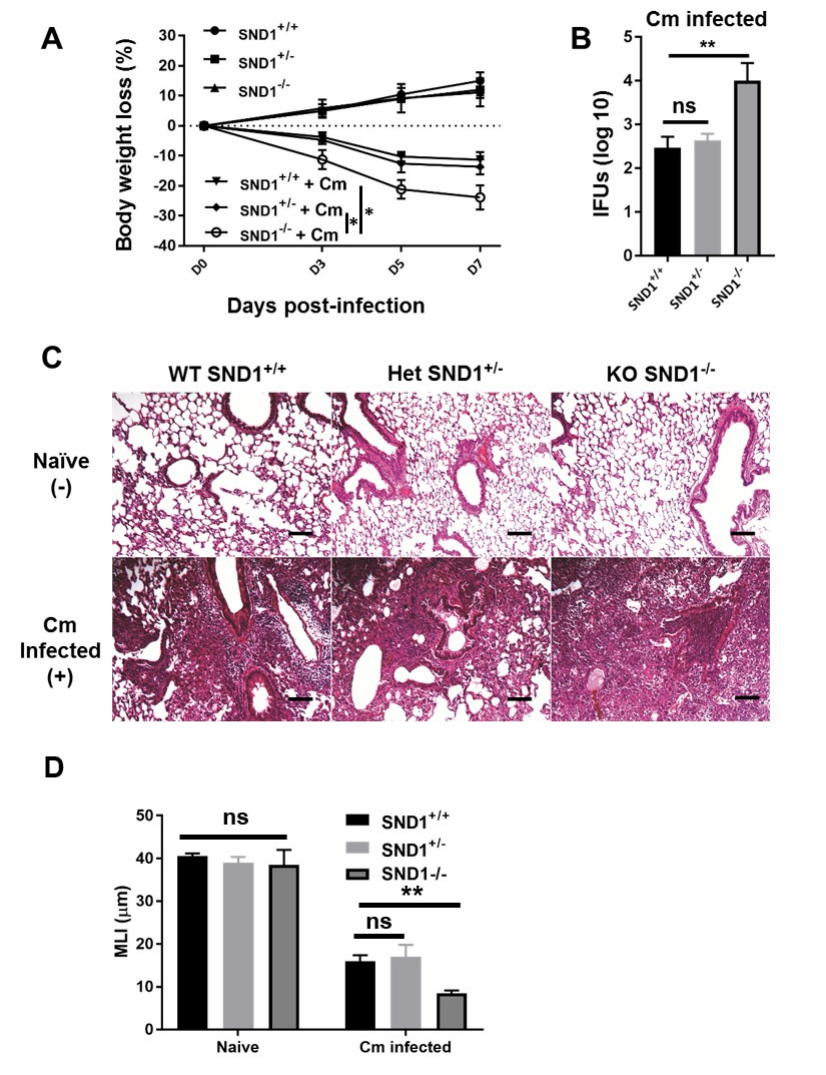
Greater body weight loss, higher bacterial growth, and more severe pathological changes in the lungs of SND1^-/-^ mice compared with that in heterozygous and control C57BL/6 mice following Cm lung infection. **(A)** SND1^-/-^ (homozygous), SND1^+/-^ (heterozygous) and wild-type control mice on a C57BL/6 background at 8-week old were intranasally infected with 1x10^3^ IFUs of Cm. Those mice without infection were used as controls. Mice were monitored every two days for body weight change. The original body weights of the three groups of mice were similar. **(B)** Mice were sacrificed on day 7 p.i., and the lungs were collected and analyzed for *in vivo* chlamydial growth, as described in *Materials and Methods*. **(C)** Lung sections were stained by H&E for histological analysis under light microscopy at day 7 (D7) p.i. One representative experiment of three independent experiments with six mice in each group is shown. Scale bar, 200 μm. (D) The MLI was calculated from the ratio of the total length of the grid lines to the total number of intersections. Results are shown as mean±SD. *, *p* <0.05; **, *p<*0.01.+ indicates as Cm infection.

To assess the role played by SND1 in host defense against chlamydial infection, a group of SND1^-/-^(homozygous), SND1^+/-^(heterozygous) and wild-type control mice on a C57BL/6 background was intranasally administered with Cm. Those mice treated with PBS without infection were used as negative controls. The results showed that following Cm infection SND1^-/-^mice suffered more severe disease compared with heterozygous SND1^+/-^ and control C57BL/6 control mice (Fig 1). The body weight loss was much greater in SND1^-/-^ mice at day 7 when the mice were killed (Fig 1A). Consistently, SND1^-/-^ mice showed significantly higher bacterial loads in the lung than SND1^+/-^ and wild-type mice at day 7 of infection (Fig 1B). The lung histological analysis showed much more tissue damage and pathological change in the SND1^-/-^ mice than in SND1^+/-^ and wild-type mice, meanwhile there was no difference in lung histological structure among the mice of SND1^-/-^, SND1^+/-^ and wild-type control mice (Fig 1C), which was further quantified by measuring the mean linear intercept (MLI), an indicator of alveolar space shown in Fig 1D. The data suggested that SND1 played a critical protective role in host defense against chlamydial lung infection.

### SND1^-/-^ mice exhibit reduced Th1/17 Cm-specific immune responses after infection

**Fig 2:**
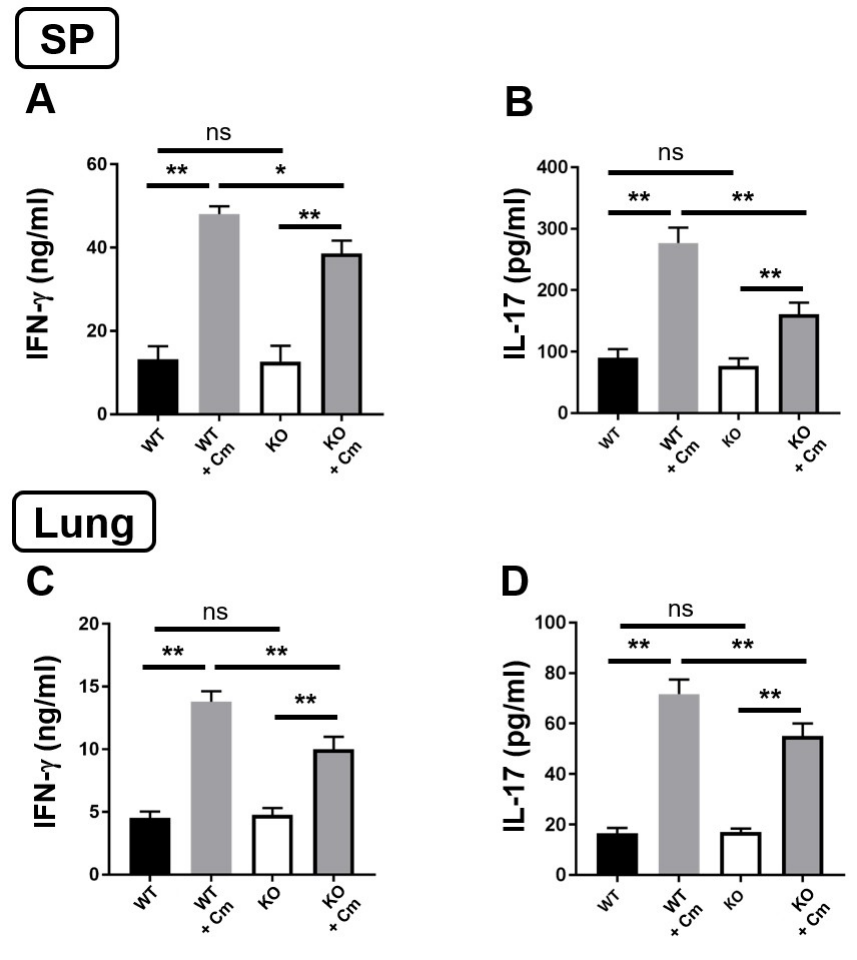
Lower Th1/Th17-related Cm-driven cytokine production in spleen and lung of SND1^-/-^ mice following Cm lung infection. Mice were infected intranasally with Cm, as described in the legend to Fig 1, and sacrificed at day 7 p.i. (**A-C**) Lower Th1 and Th17-related Cm-driven cytokine production in spleen of SND1^-/-^ mice than in WT control mice. Splenic cells were cultured with UV-inactivated EBs, as described in *Materials and Methods*. IFN-γ **(A**and IL-17 **(B)**levels in 72h culture supernatants were determined by ELISA. **(C-D)** Lower Th1 and Th17-relatedCm-driven cytokine production in lung homogenates of SND1^-/-^ mice than in WT control mice. IFN-γ **(C)** and IL-17 **(D)**levels were determined by ELISA. Data are presented as the mean±SD of per each group. One representative experiment of three independent experiments with four mice in each group is shown. *, *p* <0.05; **, *p<*0.01.+ indicates as Cm infection.

**Fig 3:**
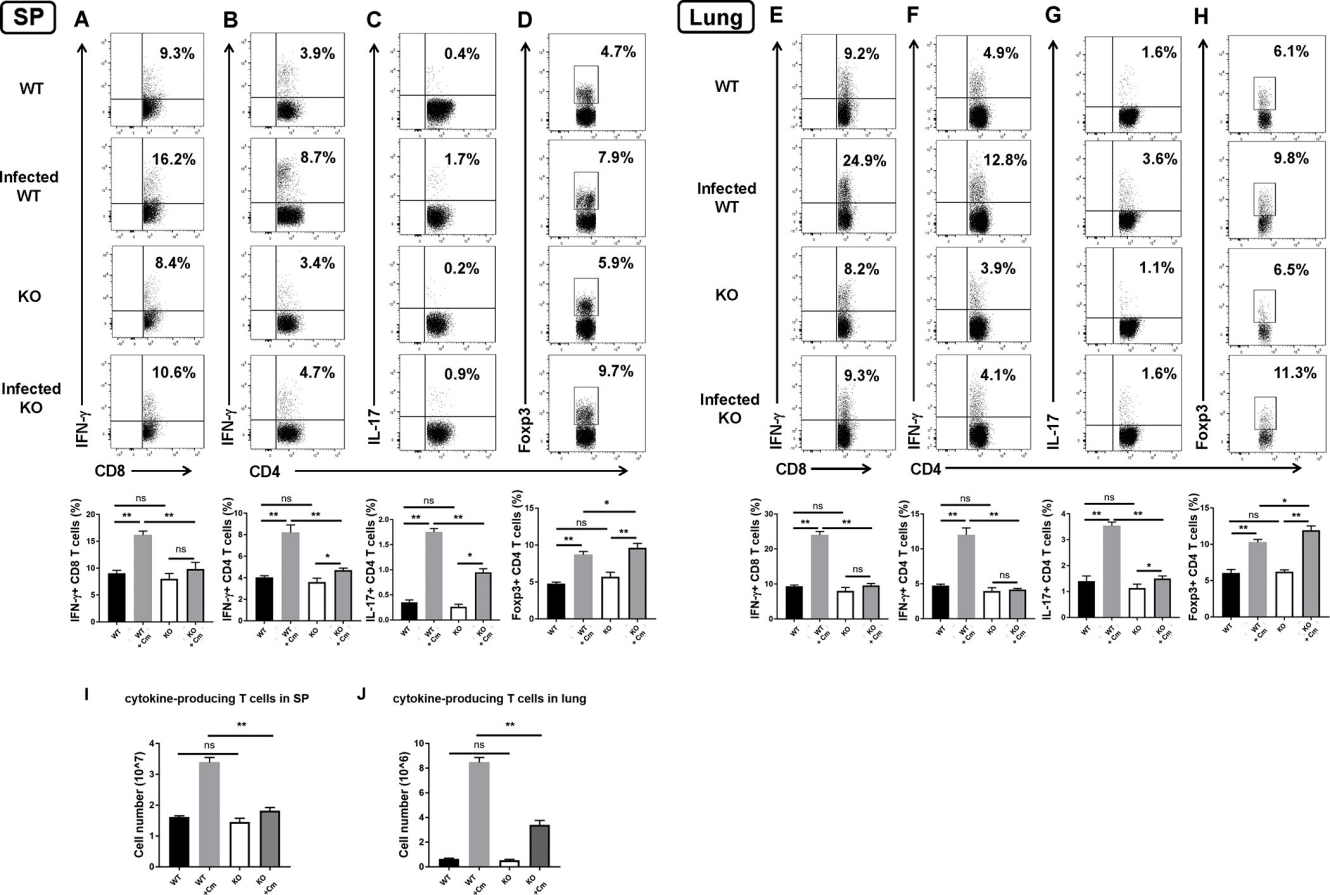
Less production of Th1/Th17 cytokine and elevated Treg response in spleen and lung of SND1^-/-^ mice compared with that of control mice following Cm lung infection. Spleen and lung collected at day 7 p.i. were analyzed for cytokine production by intracellular cytokine staining, as described in *Materials and Methods*. **(A and E)** IFN-γ-producing CD8^+^ T cells. **(B and F)** IFN-γ-producing CD4^+^ T cells.**(C and G)** IL-17-producing CD4^+^ T cells. **(D and H)** Foxp3^+^ Treg cells. Summary graphs represent IFN-γ production by CD8^+^ and CD4^+^ T cells and IL-17 by CD4^+^ T cells and Treg cells, respectively, in each group. **(I and J)** Total cytokine-producing T cells numbers in spleen and lung. Data are presented as the mean±SD of per each group. At least three independent experiments with four mice in each group were performed, and one representative experiment is shown. *, *p* <0.05; **, *p<*0.01. + indicates as Cm infection.

Because previous studies have demonstrated the association of protection with type 1 T cell responses and more severe disease severity/pathology with type 2 T cell responses against Cm infection[4,6,7], we further analyzed the Cm-driven type 1 and type 2 cytokine production by spleen and lung tissue from the infected SND1^-/-^ and wild-type mice in comparison with the sham-treated (PBS control) mice. The results showed that both spleen and lung isolated from SND1^-/-^ mice produced significantly lower levels of IFN-γ and IL-17 than wild-type mice at day 7 post-infection (Fig 2A–2D). Intracellular cytokine-staining analyses also showed significant reduction of IFN-γ- and IL-17-producing CD4^+^ and CD8^+^ T cells in both spleen (Fig 3A–3C) and lung (Fig 3E–3G) of the SND1^-/-^ mice after infection. In contrast, intracellular cytokine analysis showed significantly increased foxp3^+^ Treg cells in spleen (Fig 3D) and lung cells (Fig 3H) of SND1^-/-^ mice compared with that in the control mice after infection. Over all, under naive conditions, we found no difference in Th1/17 immune response between WT and SND1^-/-^ mice (Figs 2 and 3). In order to further support this result of cytokine profile, we quantified the total cytokine-producing T cell in spleen and lung. The results showed that both spleen and lung shared the similar cytokine-producing profile that under Cm-infected condition SND1^-/-^ mice had significantly less cytokine-producing CD4^+^T cells than wild-type mice (Fig 3I and 3J). Meanwhile, we also examined the proportion of other immune cells and found that there was no difference between WT and SND1^-/-^ mice under naive condition in CD4^+^T, CD8^+^T, B and NK cells from secondary lymphoid tissue, spleen (S2 Fig), These results showed a significant contribution of SND1 in enhancing Th1/17 cytokine responses of both CD4^+^ and CD8^+^ T cells and reducing CD4^+^Foxp3^+^ Treg cells during Cm infection.

### DCs isolated from SND1^-/-^ mice show lower IL-12 production but higher IL-10 production

**Fig 4:**
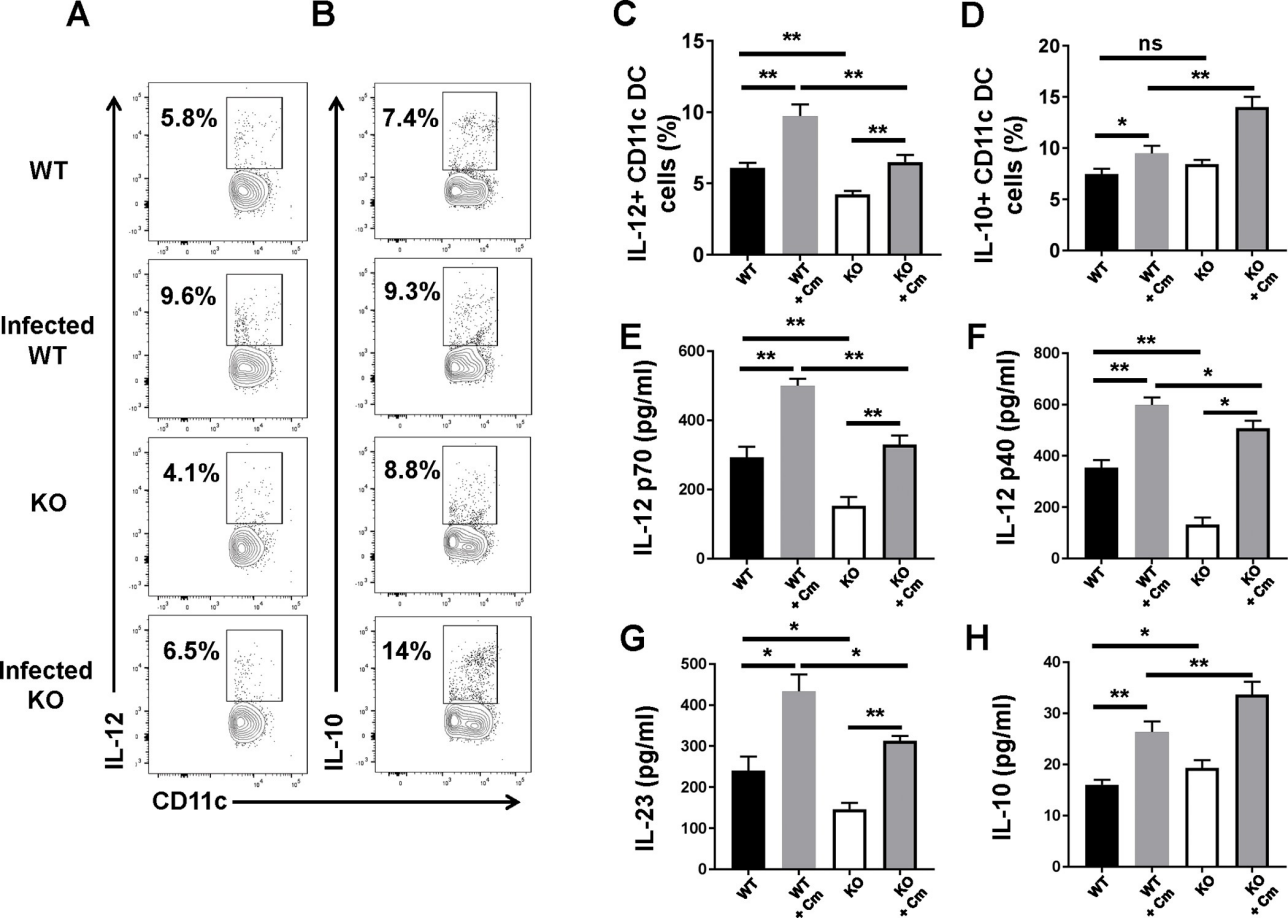
Altered cytokine production pattern of DCs in SND1^-/-^ mice compared with that of control mice following Cm lung infection. **(A and B)** Spleen collected at day 7 p.i. was analyzed for cytokine production by intracellular cytokine staining, as described in *Materials and Methods*. **(A)** IL-12-producing DCs. **(B)** IL-10-producing DCs. Summary graphs represent IL-12 **(C)** and IL-10 **(D)** production by DCs, respectively, in each group. **(E-H)** Mice were killed at day 7 p.i., and splenic DCs were isolated using MACS CD11c microbeads, as described in *Materials and Methods*. The freshly purified CD11c DCs were cultured at 5x10^5^ cells/well in 96-well plates for 72 h, followed by ELISA analysis of IL-12p70 **(E)**, IL-12p40 **(F)**, IL-23 **(G)** and IL-10 **(H)** protein levels in the culture supernatants, as described in *Materials and Methods*. One representative experiment of three independent experiments with four mice is shown. Data are shown as the mean±SD. *, *p* <0.05; **, *p<*0.01.+ indicates as Cm infection.

To explore the basis for the promoting role of SND1 in type 1 T cell responses, we examined the effect of SND1 on cytokine production by DCs during chlamydial lung infection since the cytokine profile of DCs is crucial for polarizing T-cell responses; for example, IL-12 secretion by DCs elicits Th1 responses (IFN-γ), whereas IL-23 production skews Th17 responses (IL-17). The freshly purified *ex vivo* splenic DCs were either tested by flow cytometry for intracellular cytokine of IL-12 and IL-10 or further cultured *in vitro* for 72 h to test IL-12, IL-23 and IL-10 protein production using ELISA. The intracellular cytokine-staining analyses showed that the DCs isolated from SND1^-/-^ mice expressed significantly lower levels of IL-12, but higher levels of IL-10 compared with control mice under the infected condition (Fig 4A–4D). Consistently, IL-12p40, IL-12p70, IL-23 and IL-10 levels in the culture supernatants matched the pattern of intracellular cytokine (Fig 4E–4H). Moreover, we observed under naïve condition the production of IL-12p40, IL-12p70 and IL-23 was also decreased in SND1^-/-^ mice compared to control mice, albeit to a lesser degree than infection. Overall, these data showed a decreased IL-12 production, but a higher IL-10 production pattern in DCs of SND1^-/-^ mice, especially with chlamydia infection.

### SND1^-/-^ mice exhibit impaired number and phenotype of conventional DC subsets

**Fig 5:**
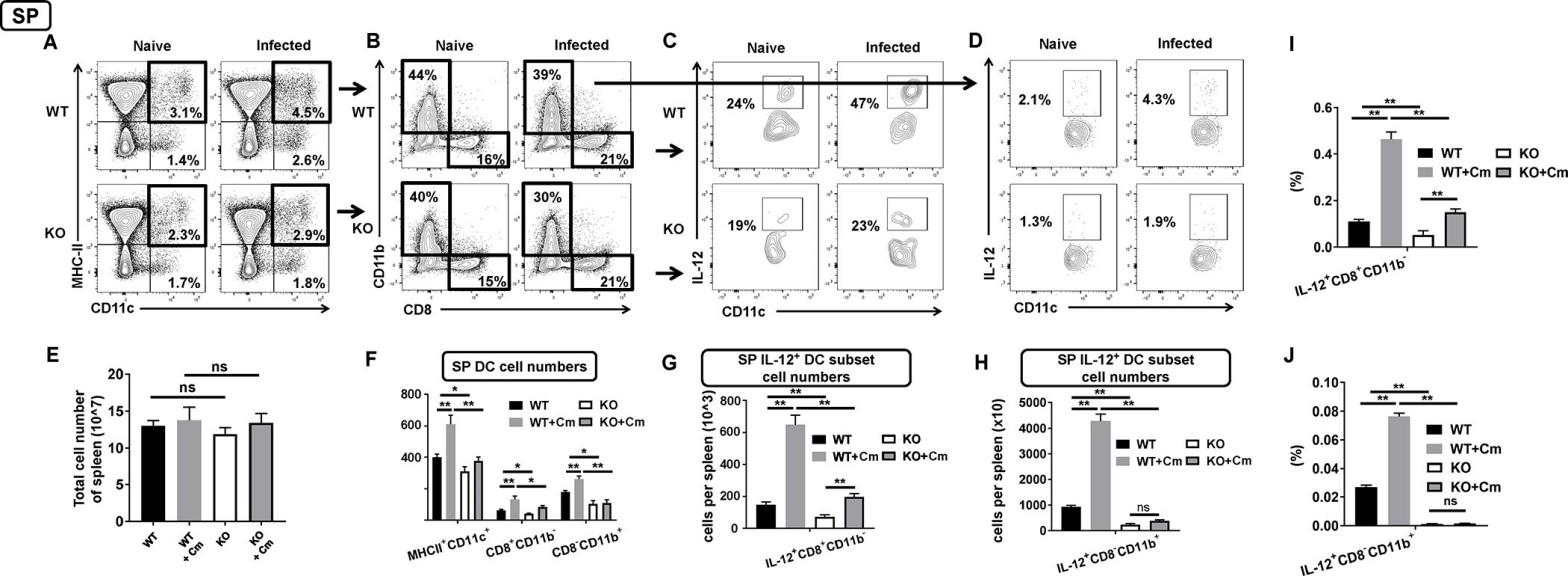
Impaired function of conventional DCs in spleen of SND1^-/-^ mice compared with that of control mice following Cm lung infection. Spleen collected at day 7 p.i. were analyzed for population of conventional DCs by surface staining, as described in *Materials and Methods*. **(A)** MHC-II^+^ CD11c^+^ conventional DC in spleen. **(B)** Two splenic DC subsets of CD8^+^ CD11b^-^ DCs and CD8^-^ CD11b^+^ DCs gated at the population of CD11c^+^ MHC-II^+^ conventional DCs. **(C)** Population of IL-12-producing CD11c^+^ DCs gated from CD8^+^ CD11b^-^ splenic DC subset. **(D)** Population of IL-12-producing CD11c^+^ DCs gated from CD8^-^ CD11b^+^ splenic DC subset. **(E)** Absolute cell number of total splenic cells in four groups. **(F)** Absolute cell number of conventional DC and two splenic DC subsets in four groups. **(G and I)** Absolute cell number and percentage of IL-12-producing CD8^+^ CD11b^-^ splenic DC subset. **(H and J)** Absolute cell number and percentage of IL-12-producing CD8^-^ CD11b^+^ splenic DC subset. One representative experiment of three independent experiments with four mice in each group is shown. Data are shown as the mean±SD. *, *p* <0.05; **, *p<*0.01. + indicates as Cm infection.

**Fig 6:**
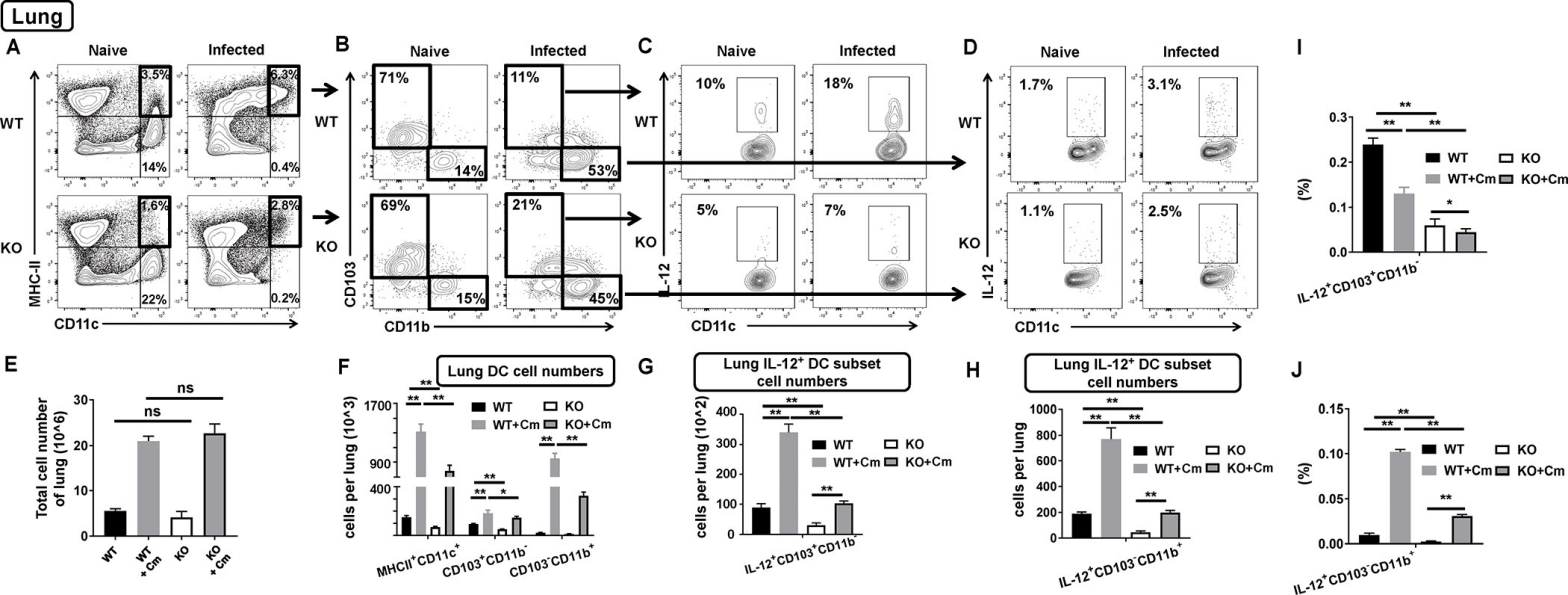
Impaired function of conventional DCs in lung of SND1^-/-^ mice compared with that of control mice following Cm lung infection. Lung collected at day 7 p.i. were analyzed for population of conventional DCs by surface staining, as described in *Materials and Methods*. (A) MHC-II^+^ CD11c^+^ conventional DCs in lung. (B) Two pulmonary DC subsets of CD11b^+^ CD103^-^ DCs and CD11b^-^ CD103^+^ DCs gated at the population of CD11c^+^ MHC-II^+^ conventional DCs. (C) Population of IL-12-producing CD11c^+^ DCs gated from CD103^+^ CD11b^-^ pulmonary DC subset. (D) Population of IL-12-producing CD11c^+^ DCs gated from CD103^-^ CD11b^+^ pulmonary DC subset. (E) Absolute cell number of total pulmonary cells in four groups. (F) Absolute cell number of conventional DCs and two pulmonary DC subsets in four groups. (G) Absolute cell number of IL-12-producing CD103^+^ CD11b^-^ pulmonary DC subset. (H) Absolute cell number of IL-12-producing CD103^-^ CD11b^+^ pulmonary DC subset. One representative experiment of three independent experiments with four mice in each group is shown. Data are shown as the mean±SD. *, *p* <0.05; **, *p<*0.01. + indicates as Cm infection.

**Fig 7:**
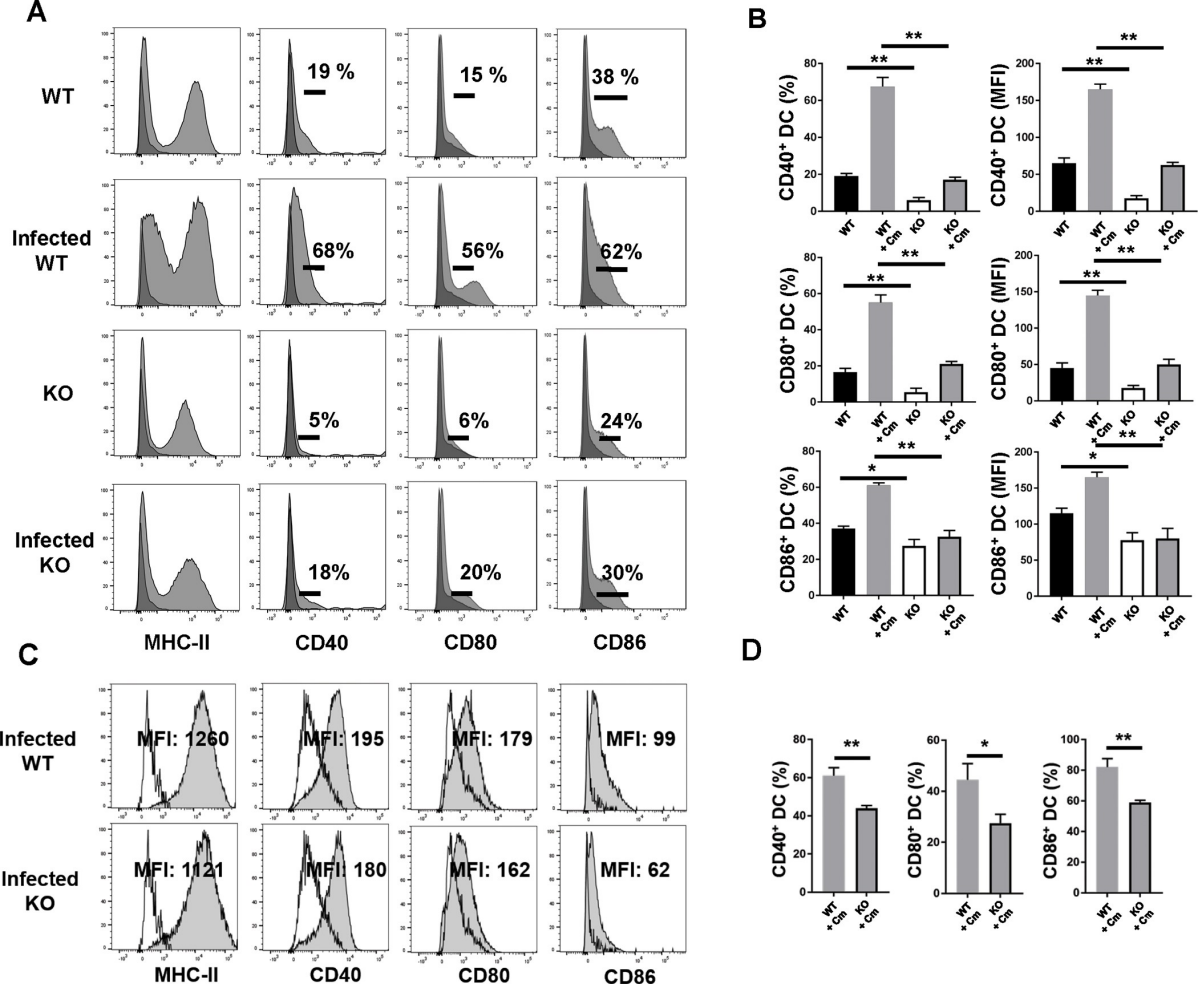
Expression of DC surface costimulatory molecules in spleen and lung of SND1^-/-^ mice compared with that of control mice following Cm lung infection. Mice were treated as described in the legend to Fig 1, and sacrificed on day 7 p.i. Splenic and pulmonary DCs were isolated using MACS CD11c microbeads, as described in *Materials and Methods*. Splenic **(A and B)** and pulmonary DCs **(C and D)** were double stained with fluorescent-conjugated anti-CD11c mAb and Abs specific for other surface markers, or with their isotype control, and were analyzed by flow cytometry. Anti-Gr-1 was used for excluding macrophages. Staining with specific Abs (grey histograms) or matched isotype Ab control (black histograms) is shown in (A). Staining with specific Abs (filled histograms) or matched isotype Ab control (black line) is shown in (C). The percentages of positive cells and the mean fluorescence intensity (MFI) for each marker are shown. One representative of three independent experiments with four mice in each group is shown. Data are shown as the mean±SD. *, *p* <0.05; **, *p<*0.01. + indicates as Cm infection.

To further investigate the influence of SND1 on subsets of DCs, we examined the changes in the frequencies of DC subsets in spleen and lung after Cm infection. We first gated on CD11c^+^ MHCII^+^ cells and then displayed by flow cytometry into CD8^+^ and CD11b^+^ subsets of spleen (Fig 5A and 5B), CD103^+^ and CD11b^+^ subsets of lung (Fig 6A and 6B), respectively. We found that the proportion of CD11c^+^ MHCII^+^ cells were decreased in spleen (Fig 5A) and lung (Fig 6A) of SND1^-/-^ mice following Cm infection at day 7. The conventional CD8^+^ CD11b^-^ DC subset in spleen, which was previously proved to be responsible for type I immunity, remained unaltered in its frequency gated from CD11c^+^ MHCII^+^ cells both under naïve and infected conditions (Fig 5B). As the spleens from wild-type and SND1^-/-^ mice before or after Cm infection were comparable in the total cell number (Fig 5E), we then calculated the absolute cell number of DC subsets and showed a decrease in numeric CD8^+^ CD11b^-^ DC subset in the spleen of SND1^-/-^ mice (Fig 5F). Further intracellular IL-12-staining analyses showed a decreased proportion of IL-12^+^ CD11c gated either from CD8^+^ CD11b^-^ DC subset or from CD8^-^ CD11b^+^ DC subset of the spleen from SND1^-/-^ mice (Fig 5C and 5D). The percentage and absolute cell number of the splenic IL-12^+^ CD8^+^ CD11b^-^ CD11c^+^ DC subset and IL-12^+^ CD8^-^ CD11b^+^ CD11c^+^ DC subset were decrease significantly in SND1^-/-^ mice after Cm infection (Fig 5G–5J). Likewise, we also examined conventional CD103^+^ CD11b^-^ DC subset in the lung, which we previously revealed its contribution to type I response to Cm infection. First, the lungs from wild-type and SND1^-/-^ mice were comparable before or after Cm infection in the total cell number (Fig 6E). Second, we calculated the absolute cell number of pulmonary DC subsets and showed a decrease in numeric CD103^+^ CD11b^-^ DC subset gated from CD11^+^ MHCII^+^ DCs in the lung of SND1^-/-^ mice (Fig 6A, 6B and 6F). Finally, in line with the result from splenic type 1 CD8^+^ CD11b^-^ DC subset, we showed a decreased proportion and absolute number of IL-12^+^ CD103^+^ CD11b^-^ CD11c^+^ DC subset of the lung from SND1^-/-^ mice (Fig 6C, 6G and 6I). Furthermore, we also found a decreased proportion and absolute number of IL-12^+^ CD103^-^ CD11b^+^ CD11c^+^ DC subset of the lung from SND1^-/-^ mice (Figs 6D, 7H and 7J). These data suggest a numeric impairment of conventional DC subsets responsive to Cm infection in SND1^-/-^ mice.

### DCs isolated from SND1^-/-^ mice show lower costimulatory molecules expression

To further understand the changes of surface markers related to DC function, we examined the expression of stimulatory molecules on CD11c^+^ DCs. Splenic SND1^-/-^ DCs showed lower expression of CD40, CD80 and CD86 molecules than splenic wild-type DCs especially at day 7 following infection both in frequency and mean fluorescence intensity (Fig 7A and 7B). Consistently, we isolated CD11c^+^ DCs from lung of infected wild-type and SND1^-/-^ mice to analyze for the same markers above. As shown in Fig 7C, compared with infected wild-type mice, Cm-infected SND1^-/-^ mice showed significantly decreased CD40, CD80 and CD86 expression on their DCs (Fig 7C and 7D). Taken together, the data suggest that SND1 deficiency in mice resulted in significantly impaired conventional DC number and phenotype, which are closely related to DC function against Cm infection.

### SND1 influences the functional ability of DC to direct T cell responses *in vitro*

**Fig 8:**
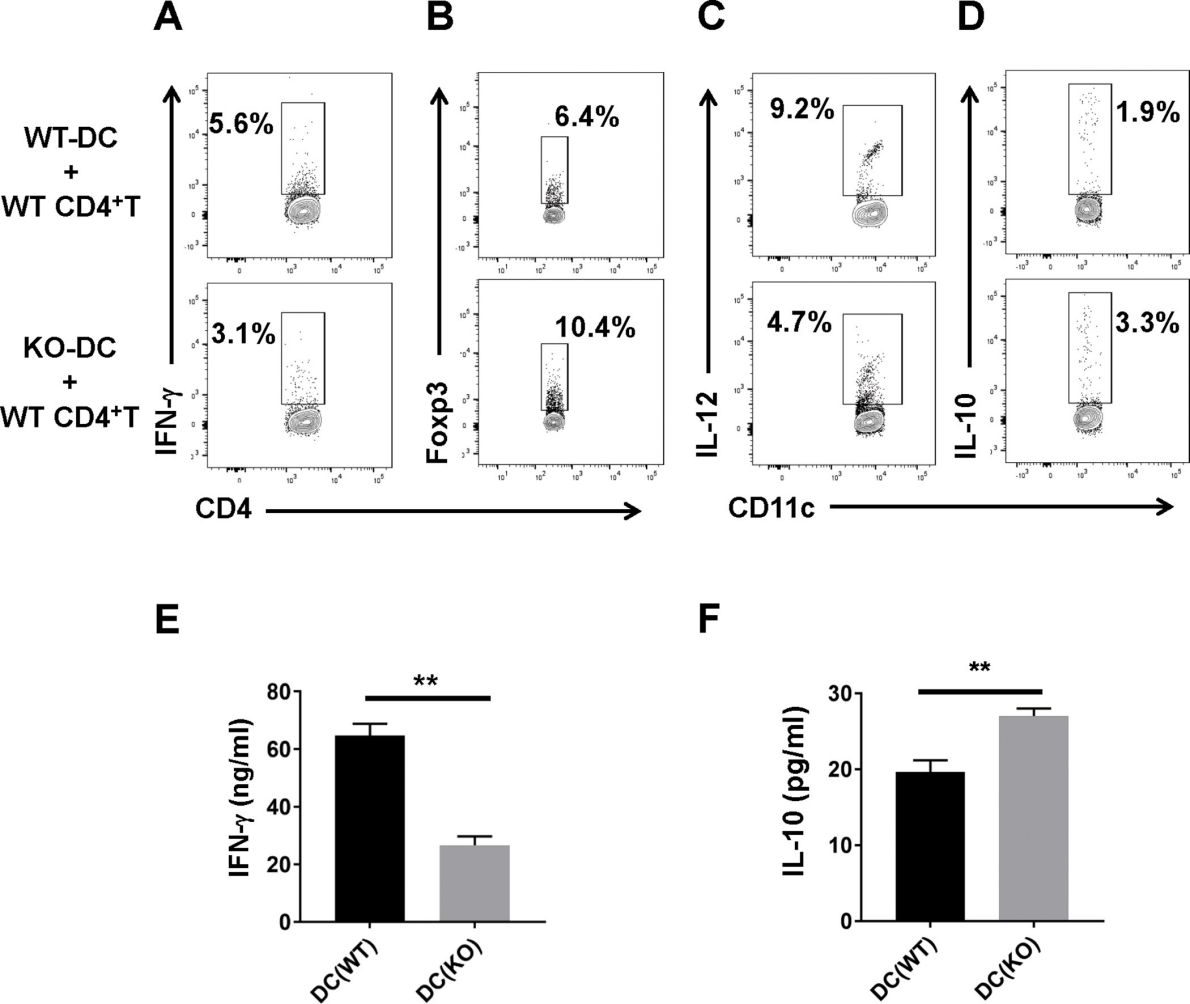
DCs from SND1^-/-^ mice show reduced Th1-promoting activity in modulating *Chlamydia*-specific T cell cytokine responses. DCs from SND1^-/-^ mice show reduced Th1-promoting activity in modulating *Chlamydia*-specific T cell cytokine responses. DCs (10^5^ cells/well) isolated from WT or SND1^-/-^ mice at day 7 p.i. were cocultured with CD4^+^ T cells (10^6^ cells/well) isolated from Cm-immunized mice in the presence of UV-killed EBs, as described in *Materials and Methods*. Cocultured cells collected at day 2 were analyzed for flow cytometry, as described in *Materials and Methods*. **(A)** IFN-γ-producing CD4^+^ T cells. **(B)** Foxp3^+^ Treg cells. **(C)** IL-12-producing DC. **(D)** IL-10-producing DC. **(E and F)** The concentrations of IFN-γ and IL-10 cytokines in the culture supernatants were measured by ELISA. One representative of three independent experiments is shown. Data are shown as the mean+-SD. *, *p* <0.05; **, *p<*0.01. + indicates as Cm infection.

To confirm whether SND1 leads to changes in DC function to direct T cell responses, we used a DC-T cell coculture system to examine the function of DC from the different groups of mice in modulating *Chlamydia*-specific T cell responses. First, we cocultured wild-type or SND1^-/-^ DCs from infected mice with *in vivo* primed CD4^+^ T cells isolated from Cm-immunized wild-type mice in the presence of UV-killed EBs to test the ability of DCs in modulating *Chlamydia*-specific T cell cytokine responses. As shown in Fig 8, DCs isolated from SND1^-/-^ infected mice showed reduced production of IL-12 (Fig 8C) and significantly reduced ability to promote *Chlamydia*-specific IFN-γ production than those from infected wild-type mice (Fig 8A and 8E) in the coculture system. In contrast, DCs isolated from SND1^-/-^ infected mice exhibited higher IL-10 production compared with wild-type DC in the coculture system (Fig 8D and 8F), skewing more CD4^+^ T cells into Foxp3^+^ Treg cells (Fig 8B). Inversely, to further exclude the possibility that the SND1 deficiency in T cells would affect T cells function, we also cocultured CD4^+^ T cells from Cm-immunized SND1^-/-^ mice with DC from wild-type mice at day 7 p.i and found that SND1 did not alter Th1 activity in this culture system (S3 Fig). Overall, these *in vitro* data demonstrated that SND1 indeed influenced the function of DCs in modulating *Chlamydia*-specific T cell cytokine patterns.

### Adoptive transfer of DCs from SND1^-/-^ mice fails to induce type 1 protective immunity against challenge infection

**Fig 9:**
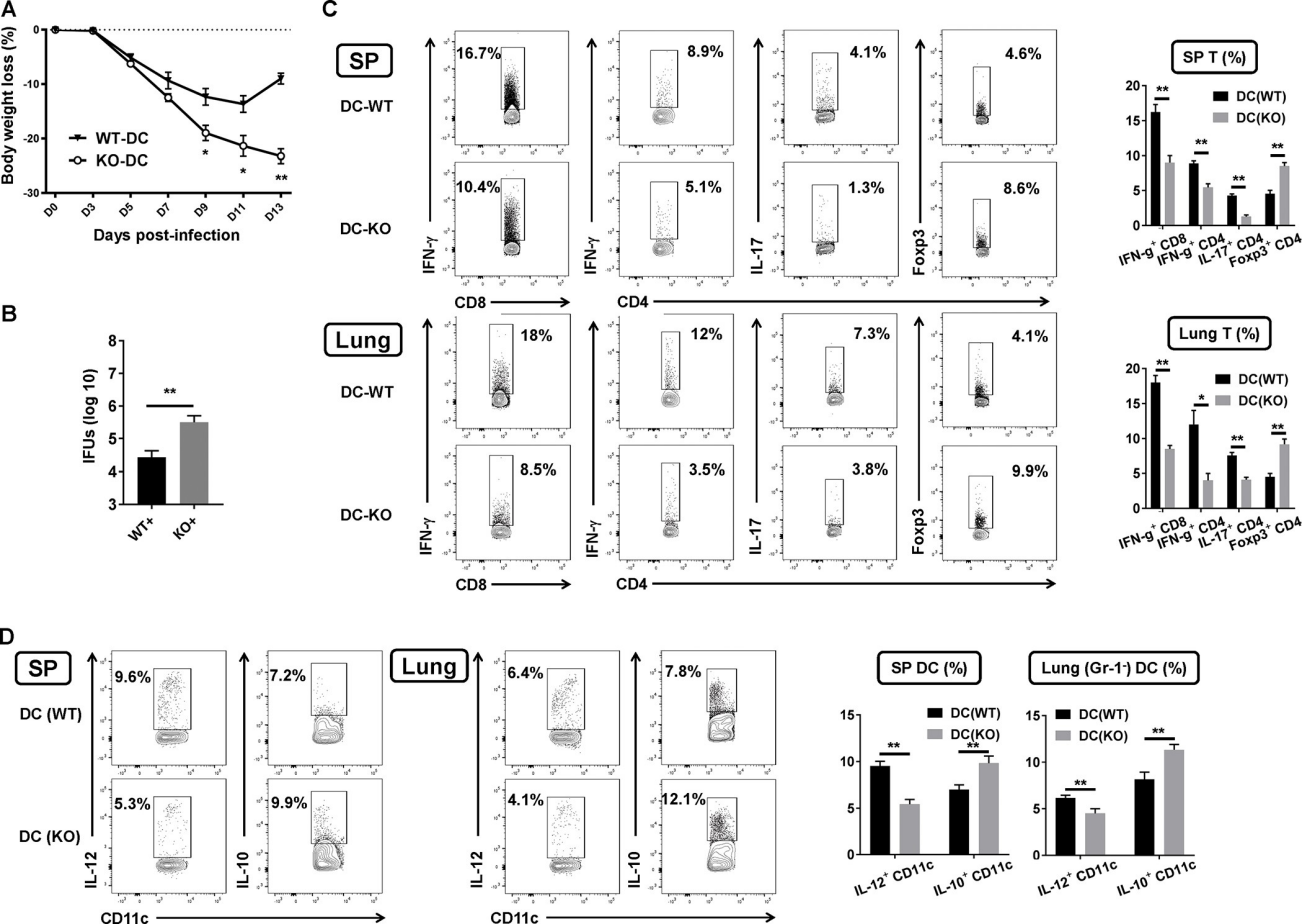
Adoptive transfer to evaluate SND1^-/-^ DC function *in vivo*. DCs isolated from the spleens of WT mice or SND1^-/-^ mice at day 7 p.i. were adoptively transferred (i.v.) to naive C57BL/6 mice recipients and intranasally challenged with 1x10^3^ IFUs of Cm at 2 h following DC transfer. Mice were killed at day 14 p.i., and the bacterial loads in the lungs and IFN-γ and IL-17 production by CD4^+^ T cells and IFN-γ production CD8^+^ T cells in spleen and lung were analyzed by intracellular cytokine staining, as described in *Materials and Methods*. **(A)** Body weight changes in two groups of mice. **(B)** Chlamydial growth in the lung. (**C)** Splenic and pulmonary cells were examined for IFN-γ-and IL-17-producing CD4^+^ and IFN-γ-CD8^+^ T cells by intracellular cytokine staining. Summary graphs represent T cells subsets in spleen and lung, respectively, in each group. **(D)** Splenic and pulmonary cells were examined for IL-12-producing and IL-10-producing DCs. Summary graphs represent cytokine-producing DCs in spleen and lung, respectively, in each group. One representative experiment of three independent experiments with six mice in each group is shown. Data are shown as the mean±SD. *, *p* <0.05; **, *p<*0.01. + indicates as Cm infection.

To further confirm the effect of SND1 on DC function *in vivo*, adoptive transfer experiments were performed to examine the ability of the DCs from different groups of mice in inducing protective immunity against challenge Cm infection. DCs were isolated from the spleens of SND1^-/-^ and wild-type mice, respectively, at day 7 p.i., and then adoptively transferred to naive syngeneic recipients by i.v. injection. The recipient mice were intranasally challenged with Cm 2hr after transfer and were killed at day 14 post-challenge. The results in Fig 9 showed that the mice that received DC isolated from Cm-infected SND1^-/-^ mice (KO-DC) exhibited much severe body weight loss (Fig 9A) and significantly higher bacterial loads (Fig 9B) than the mice with wild-type DC transfer (WT-DC), demonstrating the capacity of SND1 from DCs in generating protective immunity to challenge infection.

We further analyzed the cytokine production in the spleen and lung by intracellular cytokine staining (Fig 9C). The data showed that the mice that received DC from SND1^-/-^ mice (DC-KO) exhibited reduced levels of IFN-γ-producing CD4^+^ and CD8^+^ T cells, IL-17-producing CD4^+^ T cells, and increased levels of Foxp3^+^ Treg cells in the spleen (Fig 9C, upper panel) and the lung (Fig 9C, lower panel) compared with those that received DC from wild-type mice (DC-WT). Meanwhile, the mice that received DC isolated from the spleen and lung from Cm-infected SND1^-/-^ mice (KO-DC) exhibited reduced production of IL-12 and elevated production of IL-10 (Fig 9D) compared with those that received DC from wild-type mice (DC-WT). These findings demonstrated that SND1 contributed to the function of DC to induce protective type 1 immune responses against chlamydial infection *in vivo*.

### SND1 directly enhances IL-12 production by BMDC *in vitro*

**Fig 10:**
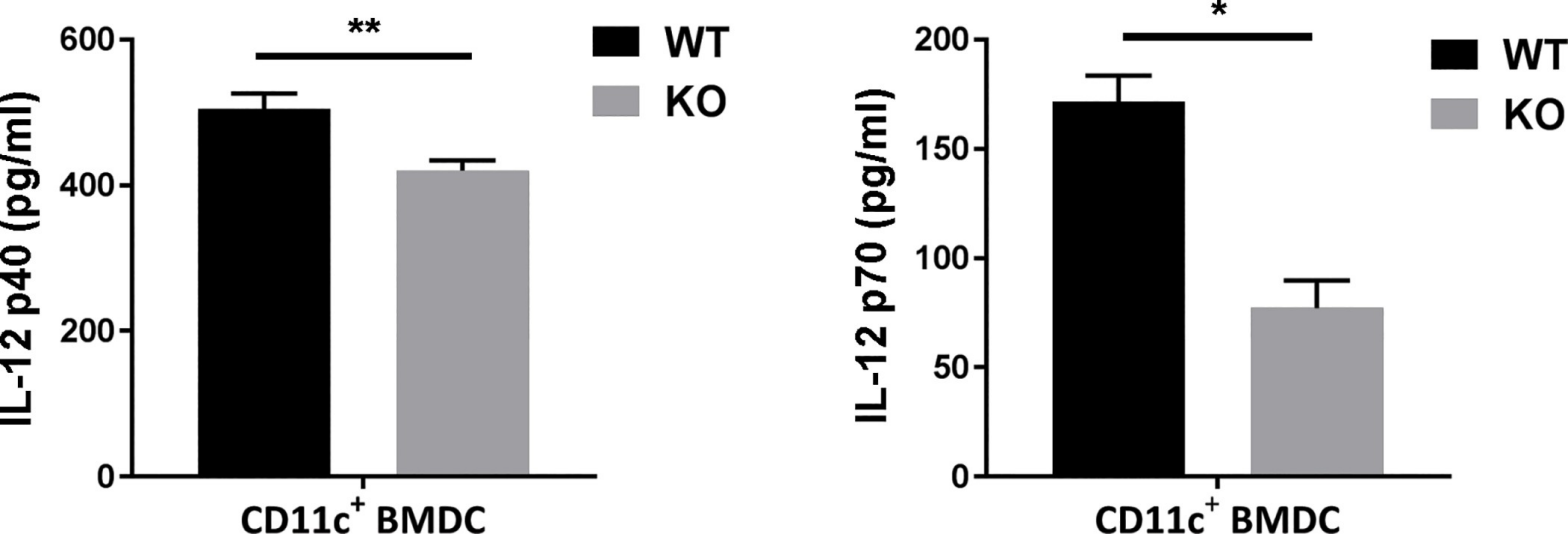
SND1 directly enhances IL-12 production by BMDC *in vitro*. Freshly isolated BM cells from WT or SND1^**-/-**^ mice were cultured in complete medium containing 20 ng/ml murine rGM-CSF, respectively, for 8 days. CD11c^+^DC were purified using MACS CD11c microbeads, as described in Materials and Methods. Purified DC were further cultured for 48 h in complete medium, and the cultured supernatants were analyzed for IL-12p40 and IL-12p70 concentrations by ELISA. One representative of three independent experiments is shown. Data are shown as the mean±SD. *, *p* <0.05; **, *p<*0.01.

The data above provided evidence on the modulating effect of SND1 on DC function during chlamydial lung infection. To further test whether SND1 could act on DC directly, we cultured BM cells isolated from WT and SND1^-/-^ mice and examined the cytokine production of BMDC. As shown in Fig 10, SND1^-/-^ DC produced significantly lower levels of IL-12p40 and IL-12p70 than those from WT BMDC. Taken together, our results demonstrate that SND1 has a direct promoting effect on DC function, which are critical for promoting type 1 T cell immunity.

## Discussion

In the present study, we demonstrated a critical role of SND1 in host defense against lung infection with Cm, an obligate intracellular bacterium. Our results showed that the SND1 mice contributed significantly to the development of Th1/17 protective immunity against the infection because the SND1^-/-^ mice showed significantly higher body weight loss, greater organism growth, and much more severe pathological changes in the lung compared with wild-type mice, which were associated with reduced IFN-γ production by CD4^+^ and CD8^+^ T cells and increased Treg cells in SND1^-/-^ mice. More importantly, we found that the SND1^-/-^ mice have impaired function of splenic CD8^+^ DC and pulmonary CD103^+^ DC, enhanced expansion of Treg cells, and reduction of Th1/17 protective immunity against the Cm infection. This report depicts a significant contribution of SND1 in Cm infection.

The most intriguing finding in the current study is the crucial modulating effect of SND1 on the cytokine production, phenotype, and, more importantly, function of DC in a real infection mouse model, and the significant effect of this modulation on T cell responses and protection *in vivo*. The modulating effect was shown by several lines of experiments. First, DC isolated from SND1^-/-^ mice exhibited significantly lower CD40/CD80/CD86 expression and IL-12 production, but higher IL-10 production than wild-type mice during Cm infection. Second, coculture of the DC isolated from SND1^-/-^ mice with primed *Chlamydia*-specific T cells showed reduced IFN-γ producing Th1 cells but enhanced capacity to induce CD4^+^ T cells into Foxp3^+^ Treg cells. These *in vitro* experiments indicated that the DCs from SND1^-/-^ mice were not efficient in inducing Th1 responses in T cell reactivation. More importantly, we showed that DC isolated from SND1^-/-^ mice, unlike those from wild-type mice, failed to generate protective Th1/17 responses and failed to protect the recipient mice against challenge infection. A similar change in pulmonary DC phenotype was also found. To our knowledge, this is the first report showing a significant modulating effect of SND1 on DC in an infection model with a real pathogen.

How does SND1 modulate DC function *in vivo*? Both direct and indirect mechanisms are most likely involved. Our data showed that under the naive condition SND1 had a direct modulating effect on the IL-12 production by means of the following DC subsets, I) splenic CD8^+^ CD11b^-^ CD11c^+^ MHCII^+^ DC (Fig 5C and 5G); II) splenic CD8^-^ CD11b^+^ CD11c^+^ MHCII^+^ DC (Fig 5D and 5H); III) pulmonary CD103^+^ CD11b^-^ CD11c^+^ MHCII^+^ DC (Fig 6C and 6G); IV) pulmonary CD103^-^ CD11b^+^ CD11c^+^ MHCII^+^ DC (Fig 6D and 6H); V) CD11c^+^ BMDC (Fig 10). Based on our data, we think it is possible that, in the beginning of infection, SND1 may influence DC function through a direct effect. Subsequently, the DC IL-12 production and type 1 immune responses promoted by SND1 may further enhance the functional ability of DC to augment Th1 and Th17 responses. Previous reports have linked SND1 to intrinsic T cell signaling[24–26], we further excluded the possibility that the SND1 deficiency in T cells would directly affect T cells function. One question remaining is why SND1 has significant effect on splenic DC. The answer remains elusive. However, because it has been demonstrated that the sources of splenic DC consist of residual DC and circulating DC[27–29], it is likely that some pulmonary DCs from infection site are able to traffic to the spleen.

Our data provide new insight into the mechanism by which SND1 plays a role in host defense against intracellular bacterial infection. SND1 was found in the present study to be critically important for the development of type 1 responses of CD4^+^ and CD8^+^ T cells and Th17 responses. Previous studies from our laboratory [30,31] and those of others [4,32] have shown that Th1/17 immune responses elicited by Cm infection are associated with protection against chlamydial infection. Our data in this study showed more severe infection and diseases in SND1^-/-^ mice, which correlated with significantly reduced Th1/17 immune responses, including lower levels of Cm-driven IFN-γ and local IL-12 production, higher levels of CD4^+^Foxp3^+^ Treg cells, and impaired function of DC. In addition to chlamydial infection, our data may provide insight into the mechanism on the role of SND1 in promoting Th1 or Th17 responses reported in other pathological models [33].

Further studies on the role of SND1 in chlamydial pathogenesis are necessary. First of all, although we have shown SND1^-/-^ mice have no alteration in the population of NK cells [34] (S2 Fig), further study on the role of SND1 in the function of these cells is worthwhile to test. This may be particularly important for understanding the mechanism by which SND1 modulates DC during chlamydial infection. Second, and more importantly, the role of SND1 at different phaces of chlamydial infection and also the host in various genetic backgrounds need to be extensively examined. Notably, the currentt study has focused on the role of SND1 in early phases of infection. In particular, we and others have shown that C3H mice strain, = remarkably susceptible to Cm infection, exhibited substantial neutrophil responses especially in the later phases of infection [27,35]. Although it is obvious that this study shows a critical role of SND1 in protection, it does not necessarily mean that SND1 at late phases of infection are also beneficial from other genetic background. Third, although Th1 response elicited by SND1 is seemed to be the dominant role for Cm clearance which might feed into Th17 response in the current model, it is worthwhile to study the potential mechanisms that whether and how SND1 is able to modulate the Th17 response independently. Finally, the slightly lower inflammatory tone of DCs in SND1^-/-^ mice was also observed under naïve condition, albeit to a lesser degree than infection, it is necessary to analyze how and why SND1 regulates DC function via a certain molecular mechanism.

In conclusion, the present study has demonstrated that SND1 plays an important role in host defense against chlamydial infection. Furthermore, the data suggest that SND1 can promote type 1 T cell-protective immunity to chlamydial infection by modulating DC function possibly through direct mechanisms. Further studies on the impact of SND1 on protective immunity and pathological responses in different phases of chlamydial infection will have potential to provide new insight into the immunobiology of chlamydia.

## Materials and methods

### Ethics statement

All mice used in this study were under the guidelines issued by the Canadian Council of Animal Care, and the research protocol was approved by the Protocol Management and Review Committee of University of Manitoba (protocol number: 15-008/3).

### Animals

Female C57BL/6 mice, 8-10-week-old, were purchased from Charles River Laboratories and housed in temperature-controlled and light/dark cycle-controlled room. SND1 knockout mice were generated at the Texas A&M Institute for Genomic Medicine (TIGM) (Texas. US) by Cre-Lox recombinase technology. See the Supplementary Information for details of the generation of the KO mice (S1 Fig). Briefly, a SND1 clone was isolated from a mouse C57BL/6 genomic DNA library. The targeting vector was designed by flanking two LoxP sites on the exon 3 of SND1 gene. The Flp-FRT recombinase technology was also used for convenient targeting vector construction and subsequent removal of unwanted neomycin selection cassette by introducing two Frt sites into the neomycin cassette within loxp flanked sequence. SND1 KO mice were maintained at a pathogen free animal care facility at the University of Manitoba in a laminar flow cabinet.

### Organism

Cm Nigg strain was used for the study. The propagation and purification of Cm were performed, as described previously[36,37]. Briefly, Cm was grown in HeLa 229 cell monolayer in Eagle’s MEM containing 10% FBS and 2 mM L-glutamine for 48 h. Infected cells were harvested with sterile glass beads, and Cm elementary bodies (EBs) were purified by discontinuous density gradient centrifugation. The purified organisms were resuspended in sucrose- phosphate-glutamic acid buffer, and stored at -80°C until used. UV inactivated EBs were used in cell culture stimulation assays for cytokine analysis[37]. The complete inactivation of EBs was confirmed by incubation of UV-treated EBs on HeLa 229 cells.

### Infection of mice

Mice were anesthetized and inoculated intranasally with 1 x 10^3^ inclusion forming units (IFU) of Cm in 40 μl final volume of PBS. Mice were killed at designated days after infection. The chlamydial growth in the lung was determined, as described[38].

### Isolation of lung

To obtain single lung cell suspensions, lungs were minced into small pieces and incubated in a solution containing collagenase XI (2 mg/ml) and DNase I (100 μg/ml) in RPMI 1640 at 37°C for 60 min. EDTA (2 mM, pH 7.2) was added at the last 5 min of incubation. Digested cells were filtered through 70-μm cell strainers, and erythrocytes were lysed using ACK lysing buffer (150 mM NH4Cl, 10 mM KHCO3, and 0.1 mM EDTA).

### DC purification and culture

The DC purification was performed, as described[36,38,39]. Briefly, spleens were aseptically removed from different groups of mice, and splenic single cell suspensions were prepared by 2 mg/ml collagenase D (Roche Diagnostics) digestion in RPMI 1640 for 30 min at 37°C. To disrupt cell aggregations and DC-T cell complexes, EDTA (5 mM, pH 7.2) was added at the last 5 min of incubation. After lysis of RBC by adding ACK buffer, CD11c^+^ DC were then isolated using the MACS (MiltenyiBiotec) system. The purity of the DC was >95%. To detect the cytokine production by DC, the freshly isolated DC were cultured with complete RPMI 1640 medium in the presence of UV-inactivated EBs (1 x 10^5^ IFUs/ml) in 96-well plates at 5 x 10^5^ cells/well for 72 h. IL-12 and IL-10 in the culture supernatants were measured by ELISA[37].

### Spleen cell cultures and cytokine assay

Spleen and draining LN cells were cultured for Cm-driven cytokine production, as described[37,40]. Briefly, single-cell suspensions were prepared and cultured at a concentration of 7.5 x 10^6^ and 5 x 10^6^ cells/well, respectively, with or without UV-inactivated Cm (1 x 10^5^ IFU/ml). The supernatants were harvested after 72-h culture, and the cytokine (IFN-γ, IL-12, IL-4, IL-10, IL-17, and IL-23) productions were measured by ELISA.

### Flow cytometry

For surface marker expression on splenic DC, freshly isolated DC were analyzed by flow cytometry, as described[6,37,41]. Briefly, the cells were double stained using fluorescent-labeled anti-CD11c and anti-MHC class II (I-A/I-E), anti-CD40, anti-CD80, or anti-CD86, or with their isotype controls (eBioscience) in a staining buffer (Dulbecco’s PBS without Ca2^+^ and Mg2^+^ containing 2% FBS and 0.09% NaN3). After staining on ice for 20 min in the dark, cells were fixed using 2% paraformaldehyde for 1 h. The data were collected using a FACS Canto-II flow cytometer and analyzed by using Cell Quest program (BD Biosciences). For analysis of the surface markers of lung, lung CD11c^+^ cells were isolated from lung single-cell suspensions using MACS CD11c column. The cells were stained with fluorescent-labeled anti-CD11c mAb, anti-Gr-1 (for excluding macrophages), and anti-CD40, CD80, CD86 or MHC II Abs, or with corresponding isotype control Abs. The gated lung DC (CD11c^+^Gr-1^-^) were analyzed for CD40, CD80, CD86 and MHC II expression.

For intracellular cytokine analysis, the single-cell suspensions of spleen, and lung were analyzed, as described[4,41]. Briefly, cells were cultured at 7.5 x 10^6^ cells/well in 48-well plates with PMA (50 ng/ml) and ionomycin (1 μg/ml) for 6 h in complete RPMI 1640 medium at 37°C. For accumulating cytokines intracellularly, 5 μg/ml brefeldinA (eBioscience) was added at the last 3 h of incubation. Cultured cells were washed twice using staining buffer and incubated with FcR block Abs (anti-16/32; eBioscience) for 15 min on ice to block nonspecific staining. Surface marker staining was performed first using fluorescent-labeled anti-CD3, anti-CD4, or anti-CD8a mAbs. Following fixation of cells for more than 30 min, cells were permeabilized with permeabilization buffer (BD Pharmingen) and stained intracellularly with fluorescent-labeled anti-IFN-γ, anti-IL-4, anti-IL-12(p70) or anti-IL-17 mAbs (eBioscience), or with corresponding isotype control Abs. Cells were washed twice with staining buffer and analyzed by flow cytometry. For foxp3^+^ T cell staining, cells were stained by foxp3 staining buffer kit (BD Pharmingen) and fluorescent-labeled anti-foxp3 mAbs.

### DC-T cell coculture

To assay the influence of DC on T cell priming and reactivation, two DC-T cell coculture systems were used, as described[8,13,41]. For examining the *Chlamydia*-specific T cell reaction by DC, CD4^+^ T cells (1 x 10^6^ cells/well) isolated from inactivated Cm-immunized mice were cocultured with the DC (5 x 10^5^ cells/well) from different groups of mice in the presence of UV-inactivated Cm (1 x 10^5^ IFUs/ml) in 96-well plates. Enriched Chlamydia- specific T cells were obtained from inactivated Cm-immunized mice, as described[41]. Briefly, mice were injected with inactivated 1 x 10^6^ IFUs of Cm i.p., and 2 wk later boosted with the same dose of infection. One week following the boosting, CD4^+^ T cells were isolated from spleen using MACS CD4 T cell column (MiltenyiBiotec), according to manufacturer’s instructions. Cell culture supernatants were harvested at 48 h for analyzing IFN-γ and IL-10 cytokines by ELISA.

### Adoptive transfer of DC and challenge infection

Freshly isolated splenic CD11c^+^ DC by a MACS CD11c column from different groups of mice at day 7 p.i. were injected into the tail vein of syngeneic naive C57BL/6 recipient mice (5 x 10^5^ DC/mouse). The recipient mice were then challenged intranasally with 1 x 10^3^ IFUs of Cm in 40 μl of PBS at 2 h following adoptive transfer. Body weights of the mice were recorded daily, and mice were killed at day 14 posttransfer of DC for analysis of bacterial loads and immune responses.

### Histopathological analysis

The lung tissues from different groups of mice were removed and fixed in 10% formalin. The tissue sections were stained by H&E, and histological changes were observed under light microscopy, as described[36,41]. The mean linear intercept (MLI) is calculated from the ratio of the total length of the horizontal grid lines to the total number of intersections for alveolar spaces in a pathological tissue image. The value of MLI was analyzed by using Image-Pro Plus[42].

### Statistical analysis

Unpaired Student’s t test was used to assay the statistical significance in the comparison of two different groups. One-way ANOVA analysis was used for analyzing data from the experiments with multiple groups. A p value less than 0.05 was considered significant.

## Supporting information

S1 Fig Construction of constitutive SND1 KO mice. The mSnd1 gene (GenBank accession number: NM_019776.2, Ensembl: ENSMUSG00000001424) is located on mouse chromosome 6. Twenty-four exons have been identified, with the ATG start codon in exon 1 and TAA stop codon in exon 24 (Transcript: Snd1-001 ENSMUST00000001460). Exon 3 was selected as conditional knockout region. Deletion of exon 3 should result in the loss of function of the mSnd1 gene. To engineer the targeting vector, homology arms and CKO (conditional KO) region were generated by PCR using BAC clone RP24-333L16 from the C57BL/6J library as template. In the targeting vector, the Neo cassette was flanked by Frt sites, and CKO region was flanked by LoxP sites. DTA will be used for negative selection. The conditional KO allele was obtained after Flp-mediated recombination and the constitutive KO allele was then obtained after Cre-mediated recombination.
(PDF)

S2 Fig A. Comparable proportion between WT and SND1-/- mice in splenic T (CD3+), NK(NK1.1+CD3-), and B(B220+CD19+) cells. B. Comparable proportion between WT and SND1-/- mice in the ratio of splenic CD4+T cells to CD8+T cells.
(PDF)

S3 Fig SND1 does not alter Th1 activity when cocolture CD4^+^ T cells from Cm-immunized SND1^-/-^ mice with DC from wild-type mice at day 7 p.i. DC (10^5^ cells/well) isolated from WT at day 7 p.i. were cocultured with CD4+ T cells (10^6^ cells/well) isolated from Cm-immunized wild-type or SND1^-/-^ mice mice in the presence of UV-killed EBs, as described in Materials and Methods. Cocultured cells collected at day 2 were analyzed for flow cytometry, as described in Materials and Methods. **(A)** IFN-γ-producing CD4 cells. **(B)** Foxp3^+^Treg cells. **(C)** IL-12-producing DC cells.
(PDF)
