# Supplementary material for: SND1 promotes Th1/17 immunity against chlamydial lung infection through enhancing dendritic cell function
Source: PLoS Pathog. 2021 Feb 26;17(2):e1009295. doi: 10.1371/journal.ppat.1009295 (PMC7946287; doi:10.1371/journal.ppat.1009295)
Supplement: S1 Fig — The mSnd1 gene (GenBank accession number: NM_019776.2, Ensembl: ENSMUSG00000001424) is located on mouse chromosome 6. Twenty-four exons have been identified, with the ATG start codon in exon 1 and TAA stop codon in exon 24 (Transcript: Snd1-001 ENSMUST00000001460). Exon 3 was selected as conditional knockout region. Deletion of exon 3 should result in the loss of function of the mSnd1 gene. To engineer the targeting vector, homology arms and CKO (conditional KO) region were generated by PCR using BAC clone RP24-333L16 from the C57BL/6J library as template. In the targeting vector, the Neo cassette was flanked by Frt sites, and CKO region was flanked by LoxP sites. DTA will be used for negative selection. The conditional KO allele was obtained after Flp-mediated recombination and the constitutive KO allele was then obtained after Cre-mediated recombination. (PDF) [file ppat.1009295.s001.pdf]

Overview of SND1 Targeting Strategy

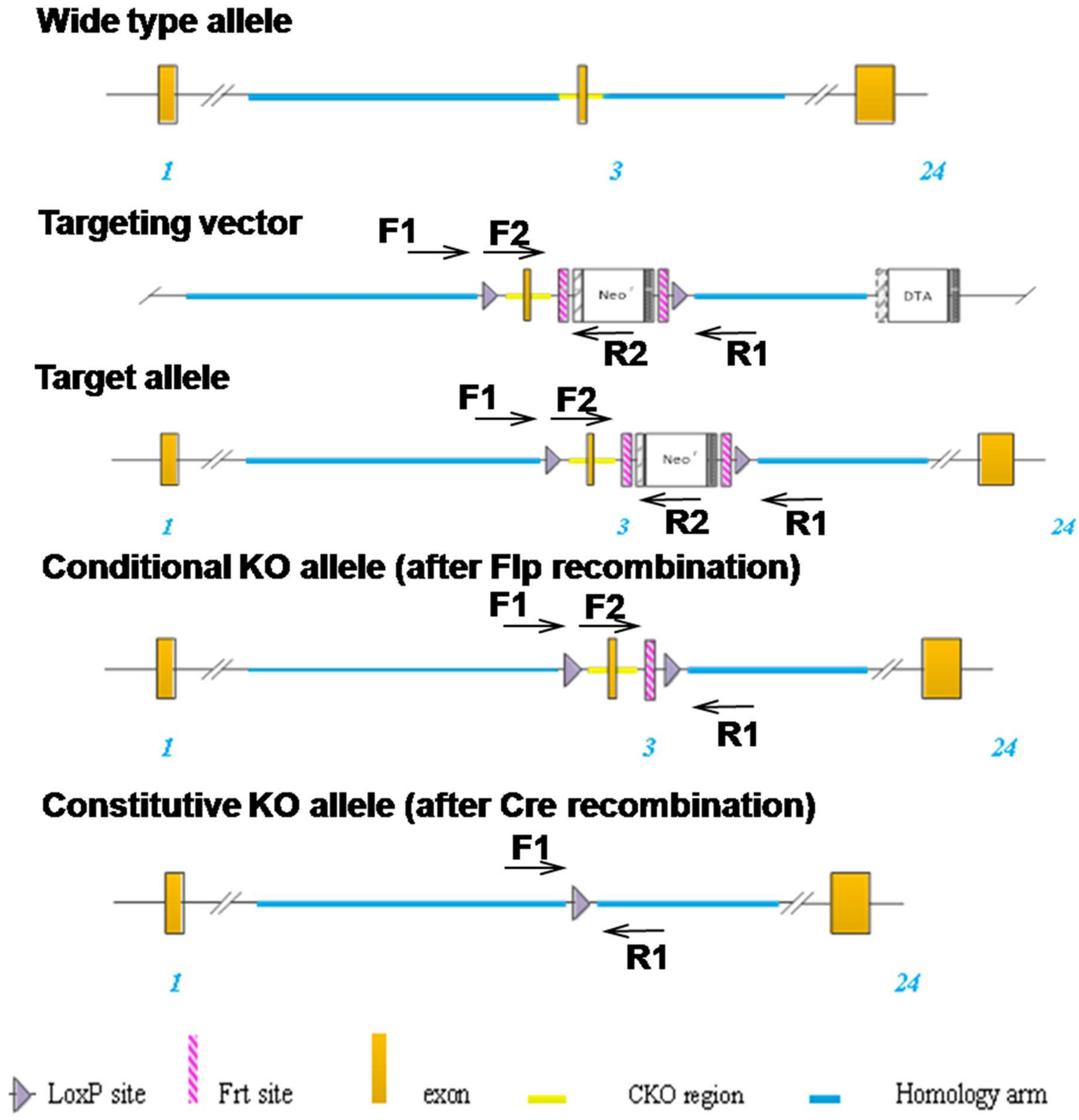

S1_Fig
