# Supplementary figures and images for: SND1 promotes Th1/17 immunity against chlamydial lung infection through enhancing dendritic cell function
Source: PLoS Pathog. 2021 Feb 26;17(2):e1009295. doi: 10.1371/journal.ppat.1009295 (PMC7946287; doi:10.1371/journal.ppat.1009295)

A

| SP(%)    | WT        | KO        | Cell surface markers |
|----------|-----------|-----------|----------------------|
| T cells  | 38 ± 2.3  | 37 ± 1.5  | CD3+                 |
| B cells  | 20 ± 2.1  | 21 ± 1.9  | B220+CD19+           |
| NK cells | 2.9 ± 0.1 | 2.8 ± 0.2 | NK1.1+CD3-           |

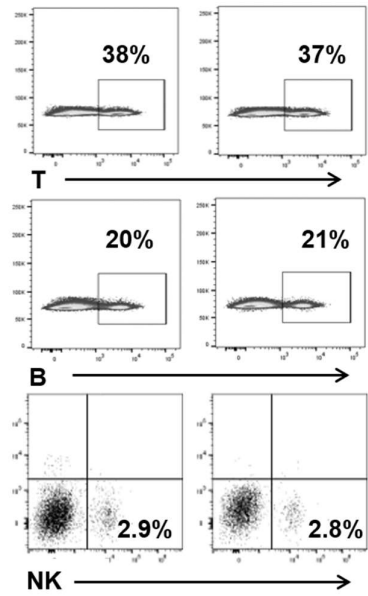

B

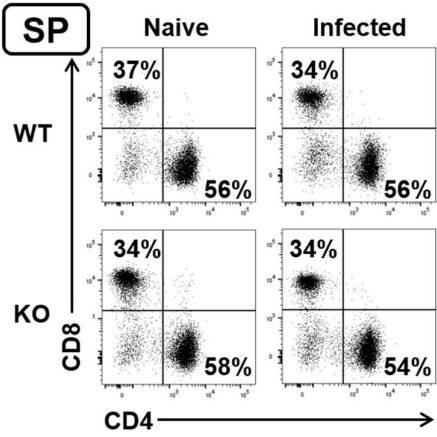

S2\_Fig

Supplement: S2 Fig — A. Comparable proportion between WT and SND1-/- mice in splenic T (CD3+), NK(NK1.1+CD3-), and B(B220+CD19+) cells. B. Comparable proportion between WT and SND1-/- mice in the ratio of splenic CD4+T cells to CD8+T cells. (PDF) [file ppat.1009295.s002.pdf]

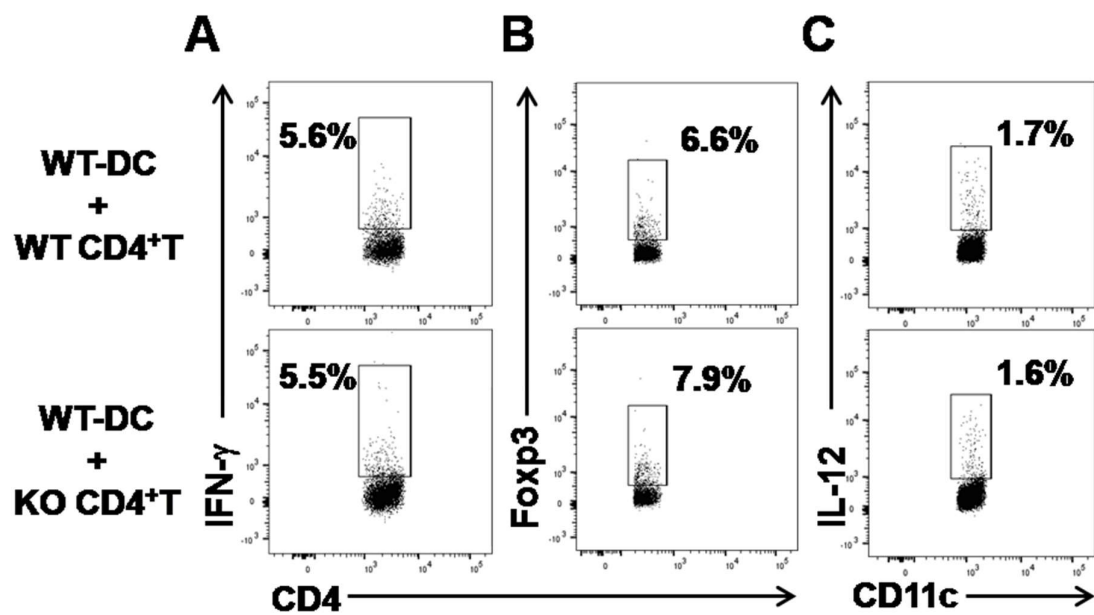

S3\_Fig

Supplement: S3 Fig — DC (105 cells/well) isolated from WT at day 7 p.i. were cocultured with CD4+ T cells (106 cells/well) isolated from Cm-immunized wild-type or SND1-/- mice mice in the presence of UV-killed EBs, as described in Materials and Methods. Cocultured cells collected at day 2 were analyzed for flow cytometry, as described in Materials and Methods. (A) IFN-γ-producing CD4 cells. (B) Foxp3+Treg cells. (C) IL-12-producing DC cells. (PDF) [file ppat.1009295.s003.pdf]
